# Synthesis and characterization of silk fibroin-MXene composite electrospun fibers for biomedical applications

**DOI:** 10.3389/fchem.2024.1471148

**Published:** 2024-12-20

**Authors:** Chengzhi Liang, Zaiwei Fan, Yudan Zhu, Yuan Cao, Jiawei Kang, Jun Tao

**Affiliations:** ^1^ Department of Orthopaedics, The Second Affiliated Hospital of Nanchang University, Nanchang, Jiangxi, China; ^2^ Department of Orthopedics, People’s Hospital of Rizhao, Rizhao, Shandong, China

**Keywords:** silk fibroin, MXene, electrospun, biocompatibility, biomedical applications

## Abstract

**Introduction:**

Two-dimensional (2D) MXene, recognized for its outstanding physical and chemical properties,has gained attention as a promising material in the biomedical field. However, its potential in tissue engineering applications remains underexplored. This study focuses on synthesizing SF-MXene composite electrospun fibers and evaluating their suitability for biomedical applications.

**Methods:**

SF-MXene composite electrospun fibers were prepared through electrospinning. The fibers were characterized using field emission scanning electron microscopy (FE-SEM), Fourier transform infrared (FTIR) spectroscopy, X-ray diffraction (XRD), mechanical testing, thermogravimetric analysis (TGA), and contact angle measurements.Protein adsorption capacity and biomineralization potential were assessed. Biocompatibility was evaluated using fibroblasts (L929) and preosteoblasts (MC3T3-E1), with alkaline phosphatase (ALP) activity measured in MC3T3-E1 cells to determine osteogenic potential.

**Results:**

The SF-MXene composite fibers exhibited well-defined morphological and structural properties, as confirmed by FE-SEM, FTIR, XRD, and TGA analyses. Mechanical testing revealed enhanced mechanical stability. The fibers showed high protein adsorption and potential biomineralization activity. Both L929 and MC3T3-E1 cells displayed high viability on the composite fibers, with significantly increased ALP activity in MC3T3-E1 cells, indicating osteogenic potential.

**Discussion:**

The findings demonstrate that SF-MXene composite fibers possess excellent structural, mechanical, and biological properties suitable for tissue engineering. The fibers’ ability to support cell viability, protein adsorption, and osteogenic activity highlights their potential in biomedical applications, particularly in bone tissue regeneration. These results suggest that MXene-based composites could be developed further for broader biomedical uses.

## 1 Introduction

In 2011, Ti_3_C_2_Tx was synthesized for the first time in the MXene family, attracting great attention since its first synthesis. MXene is characterized by atomic layers with sandwich-like layered morphology (Liang et al., 2023). Due to its high conductivity, high hydrophilicity and large specific surface area, MXene is widely used in various research fields such as energy storage, wireless communications, and biomedical applications. In the field of biomedical applications, MXene has been widely used in bioimaging, biosensors, photothermal therapies, drug delivery and antimicrobial drugs (Awasthi et al., 2020; Zhang et al., 2023). To determine whether MXene-based biomaterials are biocompatible, they must be tested for biocompatibility in general biomedical applications. Recent developments in MXene-infused biomaterials with desirable physicochemical properties have been observed. A study by Lin et al. has found that electroactive MXene hydrogels can accelerate the healing process of skin wounds by coupling ES (Electrical Stimulation) with MXene (Lin Mao et al., 2020). In a study by Ye et al., conductive Ti 2c-frozen gel enhanced cardiomyocyte function and myocardial infarction repai (Genlan et al., 2020). Hu et al. (2022) prepared electroactive hydrogels by MXene and regenerated filament factor (RSF) to promote effective bone regeneration, and demonstrated that MXene/RSF hydrogels provide a unique and promising strategy for promoting direct bone formation, regulating the immune microenvironment, and neovasculation under ES. In their study, Yu et al. developed a novel polyvinylpyrrolidone/phytic acid/MXene hydrogel which is biocompatible and can be used for SCI repair. In a rat model of complete spinal cord amputation, hydrogels significantly accelerated spinal cord regeneration by accelerating angiogenesis, myelin regeneration, axon regeneration, and calcium channel activation (Yu et al., 2023). Researchers Yang et al. developed a diabetic wound healing injectable hydrogel that combined hyaluronic acid-graft-dopamine (HA-DA) and polydopamine (PDA) (Li et al., 2022).

The electrospinning process is a general approach to preparing polymer fiber scaffolds ranging from microns to nanometers in diameter. These fibrous scaffolds mimic the natural extracellular matrix, and they are used to develop tissues, deliver drugs, coat scaffolds with polymers, and so forth in biomedical applications. There are several types of polymers that can be used in the production of nanofibers, including polycaprolactone (PCL), polylactic acid (PLA), polyglycolic acid (PGA), cellulose, chitosan, gelatin and silk fibroin (SF) (Agarwal et al., 2008). SF, as a natural fibrin, has become a promising polymer biomaterial for tissue engineering due to its extensive molecular structure, remarkable mechanical properties, controllable morphology, multifunctional processing capabilities, and surface modification options (Altman et al., 2003; Wang et al., 2006). The silk fibers of *Bombyx mori* are composed of silk fibroin (SF) coated with silk sericin (SS). The degummed silk obtained after boiling and degumming is silk fibroin (Liu et al., 2022; Wang et al., 2018). Electrospun SF fibers exhibit high surface area-to-volume ratios, high porosity, and high flexibility, which are highly desirable for biomedical and tissue regeneration applications. In the earlier studies, hydroxyapatite mineralized silk fibroin was synthesized from reclaimed silk fibroin and tussah silk fibroin for bone tissue engineering. In this study, it was found that silk fibroin composite scaffolds were biocompatible and conducive to cell attachment and growth (Andiappan et al., 2013). In a study on fibroin membrane by Liu et al. (2010), it was found that fibroin membrane is an excellent biomaterial with good cellular compatibility and provides a framework for post-trauma repair in clinical applications.

At present, electrospinning technology in addition to the preparation of single-component nanofibers, composite electrospinning nanofibers can also be modified by introducing different components to obtain the required physical, chemical and biological properties (Awasthi et al., 2020). Adding other fillers to composite nanofibers, such as *in situ* and *ex-situ* methods. Nanofiber composites are synthesized *in situ* by combining precursors of fillers with polymer solutions. A difference between *in situ* and *ex-situ* methods is that *ex-situ* methods mix the pre-synthesized particles directly into the polymer before electrospinning (Erfan et al., 2019).

By mixing different polymers with different fillers, such as carbon-containing materials (such as graphene, carbon nanotubes), nanoparticles (such as polymers and metals)[36,37], biomedical materials have enhanced electrical, chemical, mechanical, and thermal properties (Bahrami et al., 2019; Shrestha et al., 2019; Huang et al., 2018). Additionally, MXene has recently been used to modify the surface of electrospun nanofibers for biomedical applications (Mayerberger et al., 2018). As a coating nanomaterial for PLLA nanofibers, Zhu et al. proposed Ti_3_C_2_Tx MXene as a nanomaterial that provides a surface that is rich in functional groups, hydrophilic and rough, thereby supporting the adhesion and proliferation of NSCs. In this study, the MXene coating significantly increased NSC differentiation into neurons and astrocytes, providing PLLA nanofibers with multiple advantages, improving nerve regeneration in a synergistic manner (Zhu et al., 2023).

**SCHEME 1:**
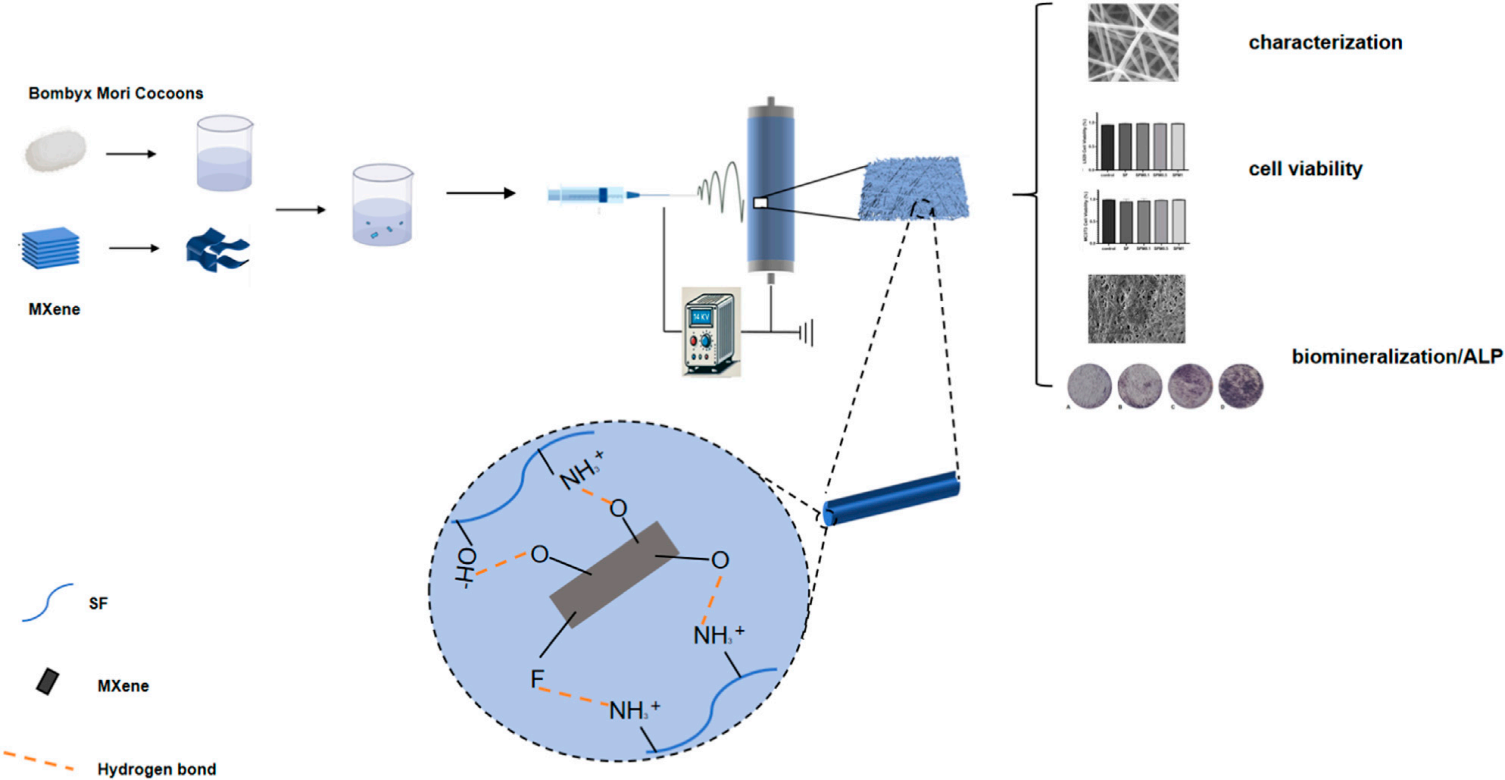
Schematic diagram of preparation of SF-MXene composites fibers.

We therefore prepared SF-MXene composite fibers by electrospinning technology using an *ex-situ* method in this study. A physicochemical and biological evaluation of synthetic composite fibers was performed to understand the potential of two-dimensional materials in biomedicine. Regenerative medicine and tissue engineering will greatly benefit in the future from the properties required for composite electrospun fiber reinforcement materials (such as hydrophilicity, mechanical properties, degradability, protein absorption, biomineralization, and cell viability) (Scheme 1).

## 2 Materials and methods

### 2.1 Materials

Ti_3_C_2_Tx MXene multilayer clay-like materials were purchased from XinXi Nanomaterial Technology Co., LTD. (Guangzhou China). *Bombyx mori* was obtained from cannongjidi (Zhejiang, China). Formic acid (CH2O2, ≥99.5%) was purchased from Aladdin (Shanghai China). Mouse embryonic fibroblast (L929) and pre-osteoblastic cell (MC-3T3) cell lines were purchased from Boya Bio (Guangzhou, China). Simulated body fluid, Cell Counting Kit-8 (CCK-8), AM/PI dual staining kit, Dulbecco’s modified Eagle’s medium (DMEM) were purchased from Solarbio Science and Technology Co., Ltd. (Beijing, China). Bovine serum albumin (BSA), and BCA protein assay kit were purchased from Nanjing KeyGen Biotech Co., Ltd. (Nanjing, China). All chemicals and solvents were of analytical grade and used without further purification.

### 2.2 Preparation of SF

The *bombyx mori* cocoons were cut into small pieces and immersed in 0.02 M Na2CO3 aqueous solution, followed by heating at 100°C for 30 min with continuous stirring to degum the cocoons. The degummed *bombyx mori* cocoons were then washed with deionized water 4-5 times to remove residual sericin on the surface and dried in a 60°C oven. The degummed silk fibers were then dissolved in a pre-prepared 9.3 M LiBr aqueous solution and stirred vigorously at 60°C to form a pale yellow solution. A semi-permeable membrane (12 kDa MWCO) was used to dialyze the pale yellow sericin-LiBr solution against deionized water at room temperature for 72 h to remove excess LiBr salt. The dialyzed solution was centrifuged at 6,000 rpm for 10 min to remove residual impurities. The sericin solution was frozen overnight at −80°C and freeze-dried to completely remove solid water, yielding a white solid sericin powder that was stored at −20°C for future use.

### 2.3 Fabrication o of electrospinning solutions

MXene of different quality was added to formic acid solution to obtain MXene of different mass fraction. After ultrasonic dispersion for 12 h, multi-layer clay Ti_3_C_2_Tx MXene was stratified into less/single layer until it was well dispersed in formic acid solution. SF (15 wt%) was then added to each prepared MXene dispersion and magnetically stirred overnight until the solution was mixed. The mass fractions of MXene in each solution were (0.1, 0.5 and 1 wt%). The other group prepared a pure SF solution by adding only SF (15 wt%) to the formic acid and slowly stirring it overnight.

### 2.4 Fabrication of SF-MXene composite electrospun fibers

The prepared electrospinning solution was sucked into a 5 mL plastic syringe using a metal capillary tube (diameter = 0.51 mm) as a needle. The high voltage power supply (Dongwen Co., LTD., Tianjin) provides 21 kV voltage and is connected to the metal needle. The syringe is connected using a digital injection pump (Ximai Technology Co., Nanjing), and the solution is set at 0.5 mL/h. The distance between the needle and the drum collector with aluminum foil on the surface (Qingdao Nuokang Technology Co., LTD., Qingdao) is 10 cm. The speed of the drum was set to 1500 rpm/min. The prepared electrospinning film was placed in anhydrous ethanol for 10 min, then washed with pure water at 37°C and dried at room temperature. The nanofiber membranes prepared with different Ti_3_C_2_Tx MXene content solutions were labeled as SF, SFM0.1, SFM0.5 and SFM1, respectively.

### 2.5 Physicochemical characterizations

The surface morphology of the electrospun fibers was observed by field emission scanning electron microscopy (FESEM, Hitachi SU8100, Japan). Fiber diameters were measured using ImageJ software (NIH, United States). The elemental composition of the electrospun fibers was confirmed by mapping with FESEM integration. Fourier transform infrared spectroscopy (FTIR; Thermo Scientific Nicolet iS20, United States), X-ray diffractometer (XRD; Panalytical Empyrean, Netherlands), has analyzed the chemical properties and crystal structure of the matrix. The hydrophobicity of electrospun fibers was measured using a contact Angle analyzer (JY-82C, China). Thermogravimetric analysis (TA Discovery TGA 550, United States) was performed at 10°C/min at 30–600°C under a nitrogen atmosphere. The mechanical strength of the nanofiber membrane was tested at a tensile speed of 5 mm/min using a universal material testing machine (MTS, China).

### 2.6 Determination of degradation rate of electrospun nanofibers

To investigate the *in vitro* degradation rate of electrospun nanofibers, samples were immersed in PBS (PH = 7.0) for 14 days at 37°C. After different time points (0, 1, 3, 5, 7, 14 days), samples were collected from PBS and excess water was removed. The weight was recorded as Wd and compared with its original weight (Wo). The rate of degradation was calculated using the following equation:

$$\text{Degradation rate}=\left[\left(\text{Wo}-\text{Wd}\right)/\text{Wo }\right]\times 100\%$$

### 2.7 Protein adsorption capacity

For the purpose of determining the synthetic nanomaterial’s adsorption efficiency, bovine serum albumin (BSA) was used as a model protein. The fiber film, cut into a 10 mm diameter circle, was placed in a test tube and incubated with 15 mL of BSA solution (2 mg/mL) at 300 rpm for 24 h. The protein concentration in the sample was measured using the BCA (bicinchoninic acid) assay, which specifically quantifies protein levels based on the colorimetric reaction between BCA reagent and proteins. The concentration of adsorbed BSA was determined by measuring the absorbance at 562 nm.

The protein concentration was calculated using the following formula:

$$\text{Protein concentration }\left(\mu g/\text{mL}\right)=\left(A\text{ determination}-A\text{ blank}\right)/\times \left(A\text{ standard}-A\text{ blank}\right)\;*\;C\text{ standard}$$

where A determination is the absorbance of the sample, A blank is the absorbance of the blank solution, A standard is the absorbance of the BSA standard, C Standard: Standard concentration, 524 μg/mL.

### 2.8 Biomineralization test

For the purpose of studying the effects of 2D MXene on SF fiber surfaces, biomimetic mineralization tests were conducted on electrospun fibers incubated with SBF. Electrospun SF and SF-MXene composite electrospun fiber pads were incubated in 5 mL SBF solution at 37°C. The fresh SBF solution is replaced every 24 h during the culture process. After incubation for 2 weeks, the sample was removed from the SBF and washed with deionized water. A FESEM analysis is conducted on the samples obtained at room temperature after they have been dried.

### 2.9 Biocompatibility assay

In order to perform the experiment, the electrospun fiber pad was sterilized in the microwave for 20 min and then transferred to a 24-well plate under ultraviolet light for 12 h. Sterile samples were rinsed with phosphate-buffered saline (pH 7.4) and co-cultured with fibroblasts/osteoblasts in DMEM.2 × 104 cell and 1,000 μL medium were co-cultured with electrospinning fiber membrane of each group, and incubated at 37°C and 5% CO2. Change the medium every 1-2 days. Three parallel control groups were set up for each sample. Incubate in the incubator according to preset time nodes (1d, 2d, 3d). According to the operation method of the CCK-8 kit, 100μLCCK-8 solution and 900 μL medium were added to each well, and incubated at 37°C for 2 h away from light. The absorbance was then measured at 450 nm using an enzyme-labeler. Cell viability is calculated by the following formula:

$$\text{cell viability}\left(\%\right)=\left(\text{measured value}-\text{blank value}\right)/\times \left(\text{control value}-\text{blank value}\right)\times 100\%$$

After the cells were incubated with the fibromembrane for 3 days, live and dead cells were labeled using a fluorescence-based live/dead cell toxicity kit according to the manufacturer’s protocol to assess cell viability. A 20-min incubation in the dark was performed on cells co-cultured with the membrane. An inverted fluorescence microscope (Nikon, Japan) was used to image staining samples after washing the cells with PBS. A software application called ImageJ was used to detect and count live cells (green fluorescence) and dead cells (red fluorescence). Cell viability is calculated using the following formula:

$$\text{cell viability}\left(\%\right)=\text{number of live cells}/\times \left(\text{number of live cells}+\text{number of dead cells}\right)\times 100\%$$

### 2.10 Alkaline phosphatase (ALP) activity

Pre-osteoblasts (MC3T3-E1) were seeded on each electrospun fiber membrane, and alkaline phosphatase (ALP) activity was detected by alkaline phosphatase staining kit after 7 days of culture. Briefly, the medium from each well was carefully removed and the cells were washed three times with PBS. Then, BCIP/NBT staining solution was added and incubated at room temperature for 2 h, and the color reaction was terminated by washing once with distilled water. Alkaline phosphatase activity was semiquantitatively analyzed using ImageJ software (NIH, United States).

### 2.11 Statistical analysis

Three parallel trials were performed for each experiment, and all data are reported as mean ± SD. All experimental results were compared using one-way ANOVA, and the data were statistically analyzed using GraphPad Prism 8.0 software. A statistical difference of P < 0.05 was considered significant. (*P < 0.05, **P < 0.01, ***P < 0.001, and ****P < 0.001)

## 3 Results and discussion

### 3.1 Physiochemical properties

**FIGURE 1:**
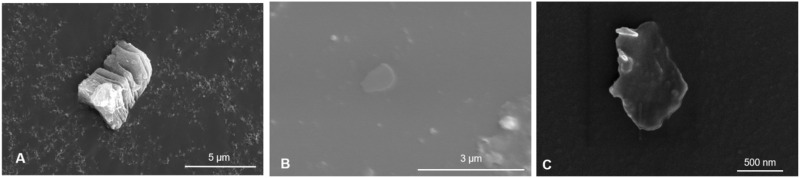
Multi-layer clay Ti_3_C_2_Tx MXene SEM image **(A)**. Ultrasonic dispersed few/single layer Ti_3_C_2_Tx MXene SEM image **(B, C)**.

Few/single-layer MXene nanosheets were obtained by ultrasonically exfoliating clay-like multilayer Ti_3_C_2_Tx MXene. Scanning electron microscope (SEM) images of the multilayer Ti_3_C_2_Tx MXene showed a distinct multilayer nanosheet structure (Figure 1A). After 12 h of ultrasonic dispersion, the nanosheets exhibited an obvious few/single-layer structure under the SEM (Figures 1B, C).

**FIGURE 2:**
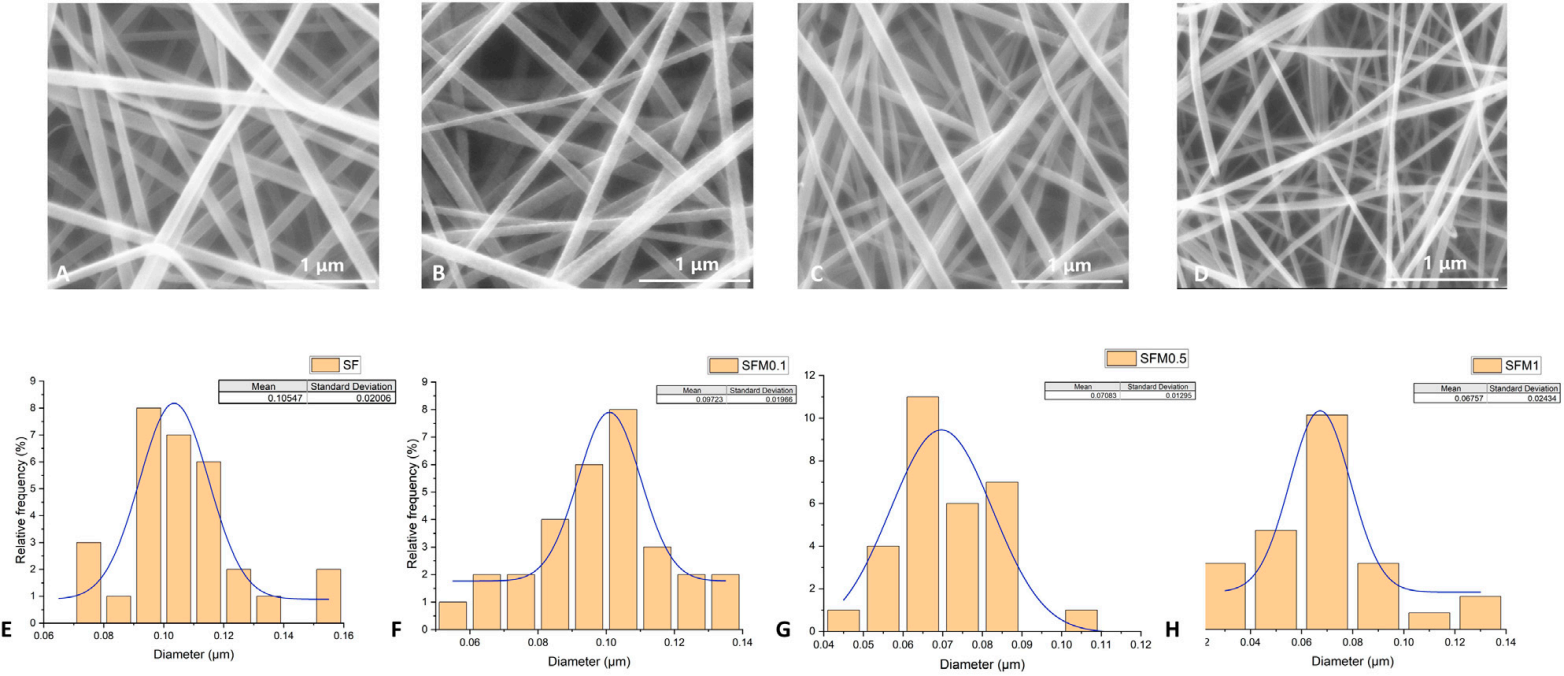
SEM image and fiber diameter distribution of SF electrospinning fibers **(A, E)**. SEM image and fiber diameter distribution of SF-MXene composite electrospinning fibers (0.1 wt%, 0.5 wt% and 1 wt%) **(B–D, F–H)**.

**FIGURE 3:**
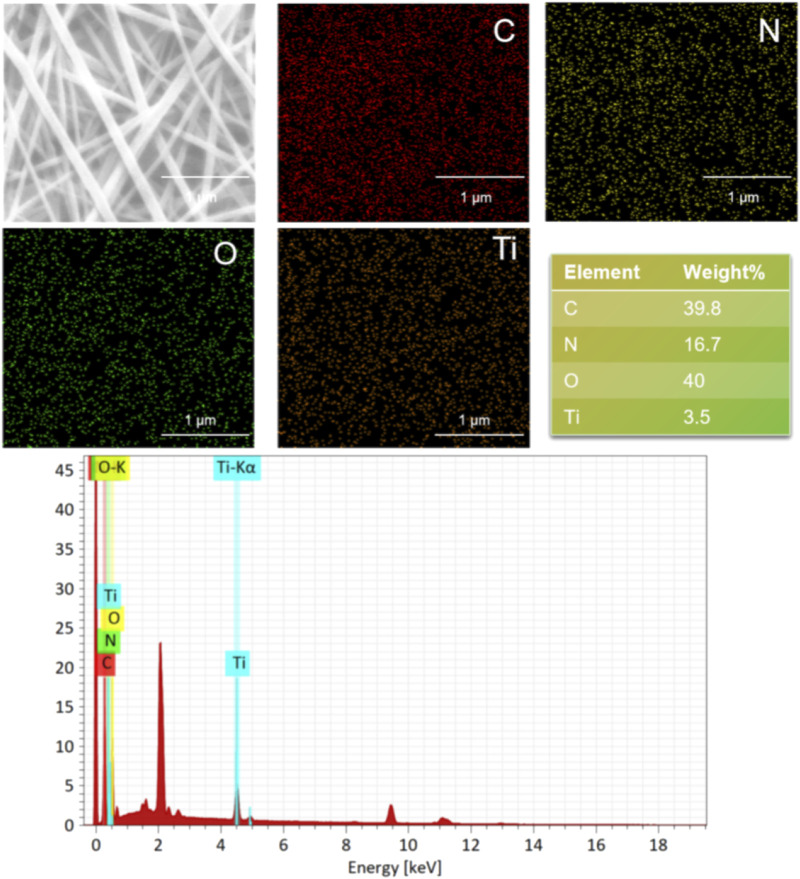
Elemental mapping and EDS (bottom) of SF-MXene (1 wt%) composite electrospun fibers.

The surface morphologies of SF and SF-MXene composite electrospun fibers doped with different weight percentages of Ti_3_C_2_Tx MXene are shown in Figures 2A–D. As the content of Ti_3_C_2_Tx MXene increased, the fiber morphology of the composite fibers did not change significantly. Compared with SF fibers, the diameter of the composite fibers decreased after the incorporation of Ti3C2Tx MXene, and as the MXene content increased, the diameter gradually decreased. The histograms of fiber diameters for each group are shown in Figures 2E, F. The average fiber diameter decreased with increasing MXene content: SF fibers had a diameter of 0.105 ± 0.02 μm, 0.1 wt% MXene was 0.097 ± 0.02 μm, 0.5 wt% MXene was 0.071 ± 0.01 μm, and 1 wt% MXene was 0.068 ± 0.02 μm. Statistical analysis using ANOVA was performed to evaluate the significance of these changes. The results showed that the fiber diameter significantly decreased with increasing MXene content, with p-values of <0.0001, indicating a statistically significant difference between the groups. This change is likely due to the inherent conductivity of MXene, which increases the conductivity of the spinning solution, leading to a more pronounced stretching of the Taylor cone under the same electric field strength. After doping, the fiber surface remained smooth. In addition, the presence of Ti elements in the SF-MXene composite fibers was confirmed by SEM-EDS, indicating that Ti_3_C_2_Tx MXene had been incorporated into the fibers, as shown in Figure 3.

**FIGURE 4:**
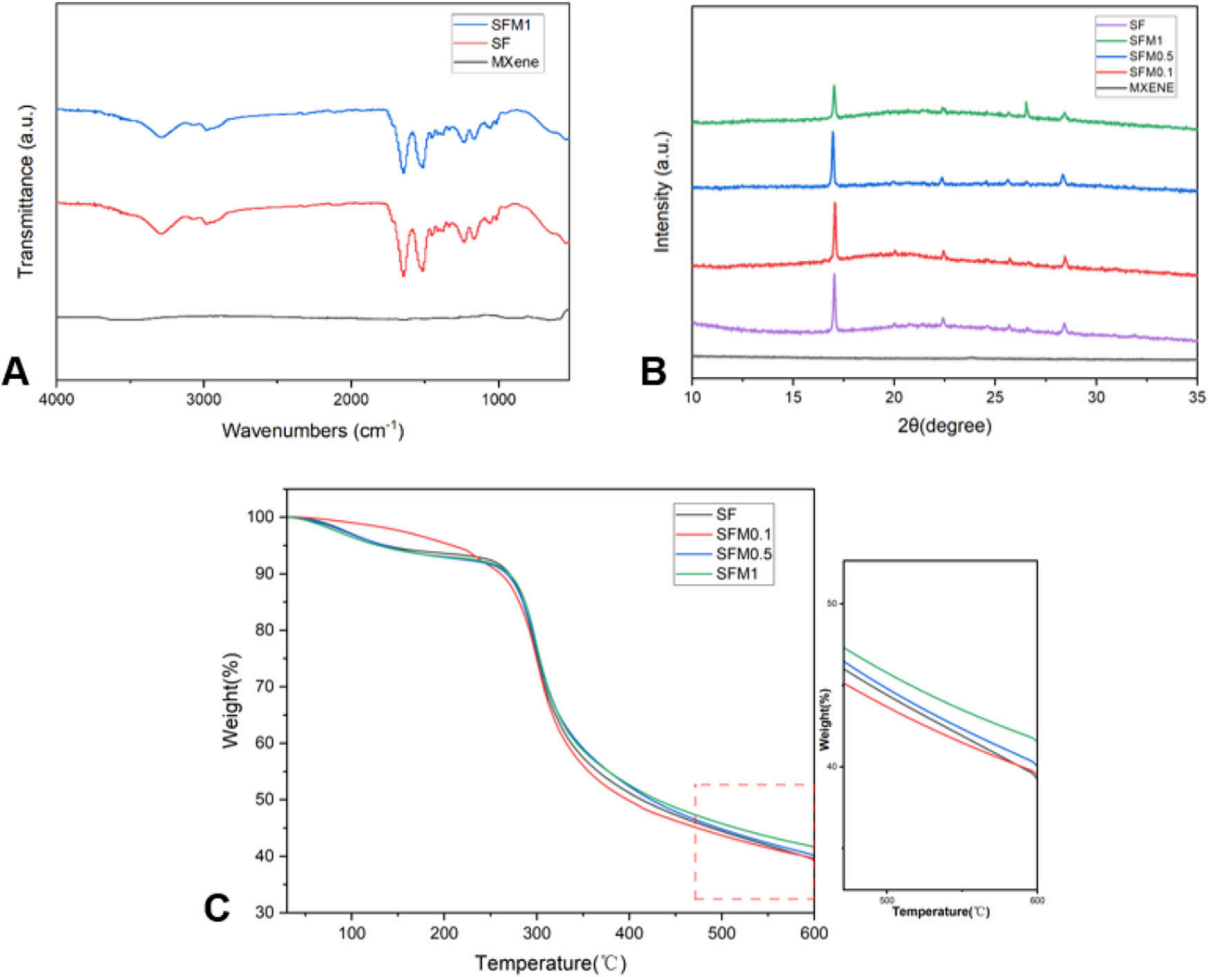
FTIR **(A)** for SF, SF-MXene (1 wt%) composites and MXene powders. XRD **(B)** of SF, MXene powder and SF-MXene (0.1 wt%, 0.5 wt% and 1 wt%) composites. TGA **(C)** of SF, SF-MXene (0.1 wt%, 0.5 wt% and 1 wt%) composites.

As shown in the FTIR spectra (Figure 4A), the SF fibers exhibit distinct absorption peaks at 3,288 cm^-1, 2,986 cm^-1, 1,645 cm^-1, 1,516 cm^-1, 1,233 cm^-1, 1,168 cm^-1, 1,055 cm^-1, and 542 cm^-1. The broad peak at 3,288 cm^-1 corresponds to the O-H stretching vibration in silk fibroin. The absorption peak at 2,986 cm^-1 is attributed to the C-H stretching vibration. The absorption peaks at 1,645 cm^-1, 1,516 cm^-1, and 1,233 cm^-1 are characteristic bands of amide I, amide II, and amide III of the protein in silk fibroin, respectively. The peaks at 1,168 cm^-1 and 1,055 cm^-1 are due to the C-OH and C-O stretching vibrations. These characteristic absorption peaks are consistent with the typical structure of silk fibroin (Yoshimizu and Asakura, 1990). The presence of a prominent broad absorption band at 3,400 cm^−1^ in MXene indicates strong external water absorption on its surface [51]. In SF-MXene composite electrospun fibers, the peak intensities at 542 cm^-1^ and 3,288 cm^-1^ are reduced. This attenuation is attributed to the formation of hydrogen bonds between MXene and silk fibroin molecules, which weakens the stretching vibrations of C-N and O-H bonds. The narrowing and reduced intensity of the characteristic peak at 1,645 cm^-1^ suggest that the incorporation of MXene destabilizes the β-sheet structure, leading to a transition towards an α-helix structure.

We further conducted XRD tests on various nanofiber groups, as shown in Figure 4B. Previous studies on silk fibroin (SF) have indicated that the main diffraction peaks of silk fibroin I structure appear at 12.2°, 19.7°, 24.7°, and 28.2°, while those of silk fibroin II structure are located at 9.1°, 18.9°, 20.7°, and 24.3° (Ding et al., 2023; JX et al., 2022). In this study, the XRD curve of the silk fibroin membrane exhibited typical peaks at 19.7°, 20.7°, 24.7°, and 28.2°, providing strong evidence for the presence of silk fibroin I structure along with a small amount of silk fibroin II structure. As the Ti_3_C_2_Tx MXene content increased, the diffraction peak at 26.6°in the composite fiber XRD curve became more prominent. This might suggest that the introduction of Ti_3_C_2_Tx MXene altered the crystal structure of the composite material. MXene possesses abundant surface functional groups (such as -OH and -F), which could interact with amino or carbonyl groups in silk fibroin through hydrogen bonding or electrostatic interactions, enhancing the hydrophilicity and structural stability of the composite material and thus inducing changes in the structure of silk fibroin. This structural change might lead to a transition from a β-sheet structure to an α-helix structure, manifested as an enhancement associated with the 26.6°peak in the XRD pattern. Additionally, with increasing Ti_3_C_2_Tx MXene content, the diffraction peak at 17.6°in the silk fibroin-MXene (SFM) curve gradually decreased. This phenomenon could be attributed to the overlap of the characteristic peak of Ti_3_C_2_Tx MXene at 14.5°(004) with silk fibroin, resulting in a left shift of the peak position. This shift indicates an increase in the unit cell constant, suggesting the successful incorporation of Ti_3_C_2_Tx MXene into the silk fibroin structure, which may have led to microscopic adjustments in the structure of the composite material.

Thermogravimetric analysis (TGA) was performed on SF and SF-MXene composites, and the corresponding TGA curves are presented in Figure 4C. Both SF and SF-MXene composite fibers exhibited two distinct stages of mass loss during thermal degradation. The first stage was attributed to the loss of moisture from the fiber membranes. The second stage, occurring between 270°C–330°C, corresponded to the degradation of SF itself. This mass loss was due to the decomposition of SF polymer chains, primarily through the loss of volatile components such as small molecules and degradation products. Interestingly, as the weight percentage of Ti3C2Tx MXene increased, the residual mass of the composite fibers also increased. This suggests that MXene enhances the thermal stability of SF fibers, which may be related to the high thermal conductivity and stability of MXene nanoparticles. The increased residual mass confirms the successful incorporation of MXene into SF fiber membranes at different weight percentages (Z et al., 2022; Liu et al., 2021). The presence of MXene particles serves as a thermal stabilizer, reducing the overall mass loss during thermal degradation.

**FIGURE 5:**
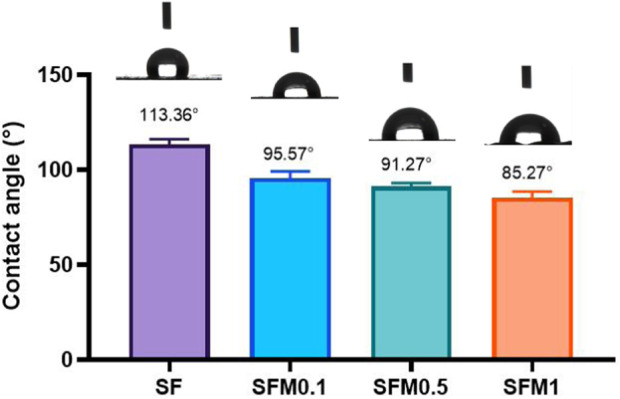
Wettability test of SF and SF-MXene(0.1 wt%, 0.5 wt% and 1 wt%) composite electrospun fibers.

The hydrophilicity of materials plays a crucial role in their interaction with cells, as it directly affects the material’s ability to adsorb proteins and interact with the biological environment. To evaluate whether the addition of MXene nanoparticles improves the hydrophilicity of composite electrospun fibers, we measured the water contact angle (WCA), as shown in Figure 5. The pure SF fibers exhibited a contact angle of 113.36° ± 2.84°, indicating their hydrophobic nature. However, with increasing proportions of Ti3C2Tx MXene, the wettability of the composite fibers improved, evidenced by a gradual decrease in the contact angle: SFM0.1 had a contact angle of 95.57° ± 3.63°, SFM0.5 showed 91.27° ± 1.83°, and SFM1 demonstrated 85.27° ± 3.16°.The enhanced wettability with increasing MXene content can be attributed not only to the hydrophilicity of hydroxyl and oxygen-containing functional groups on the MXene surface (Naguib et al., 2011) but also to the increased surface roughness and porosity of the electrospun fibers. As the MXene content increases, the fiber diameter decreases, leading to an increased specific surface area of the fiber mat. This increase in specific surface area creates more contact points between the fiber mat and water molecules, thereby improving its wettability. Furthermore, the increased porosity aids in capillary action, allowing the fibers to absorb water more effectively, further enhancing their hydrophilicity. Thus, the increased MXene content improves not only the surface chemical properties of the fibers but also their physical structure, both of which contribute to the observed enhancement in wettability and hydrophilicity.

**FIGURE 6:**
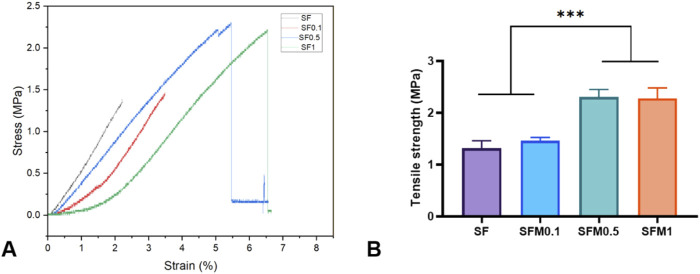
**(A)** Stress-strain curves, **(B)** Tensile strength of the SF and SF-MXene (0.1 wt%, 0.5 wt% and 1 wt%) nanofiber samples. (***P < 0.001).

Good mechanical properties have better stability and ductility, which can play a better role in biological tissue engineering. To elucidate the effect of adding Ti_3_C_2_Tx MXene on the mechanical properties of the scaffolds, the tensile strength of various nanofibrous scaffolds was measured. Figure 6 shows the stress-strain curves of composite fibers without and with 0.1wt%, 0.5wt%, and 1wt% MXene. With the increase of MXene content, the mechanical strength of composite fiber at 0.5wt% and 1wt% was significantly higher than that of pure SF and 0.1wt%. This may be due to the interaction between MXene surface rich oxides or fluorides and a large number of amino acid residues in SF, especially hydroxyl groups and amino groups, through hydrogen bonding. Thus, the mechanical properties of the composite fibers are improved.

### 3.2 Degradation rate of electrospun nanofibers

**FIGURE 7:**
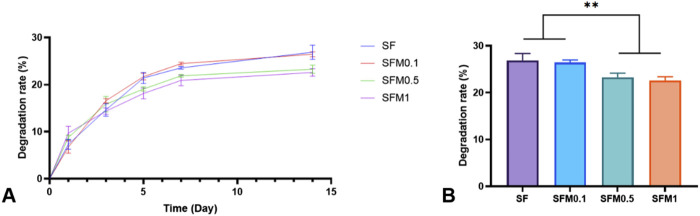
**(A)** Degradation curves within 14 days, **(B)** Degradation at 14 days of the SF and SF-MXene (0.1 wt%, 0.5 wt% and 1 wt%) nanofiber samples. (**P < 0.01).

The degradation performance of biomaterials is also one of the important characteristics of their applications, and excellent degradation performance will be conducive to their wider application in more scenarios. In this study, we investigated the degradation properties of SF and electrospun fiber membranes with different contents of Ti_3_C_2_Tx MXene within 2 weeks. As shown in Figure 7, panel A shows the degradation rate of each group’s fibrous membrane at different time points within 14 days, and panel B shows the degradation rate of each group’s fibrous membrane at 14 days. As can be seen from the degradation curve in FIG. A, the degradation rate of all samples reached the maximum at 5–7 days and tended to be flat at about 14 days. The results of panel B show that the degradation rate of the samples with high content of MXene addition (0.5wt% and 1wt%) is significantly lower than that of the samples with low content of mxene addition (0wt% and 0.5wt%). This may be due to the introduction of mxene nanoparticles, the fiber structure becomes compact due to hydrogen bonding, electrostatic interaction and other forces, the mechanical strength increases, and the degradation rate phase is relatively slow.

### 3.3 Protein adsorption capacity

**FIGURE 8:**
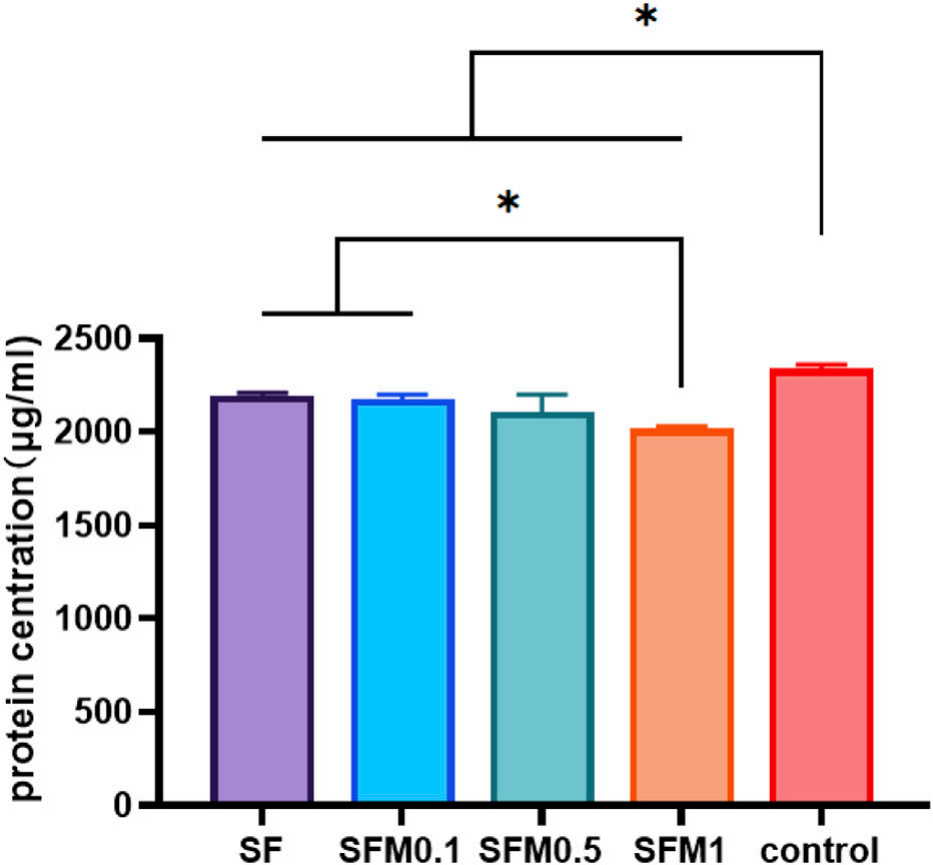
Protein adsorption capacity test of SF and SF-MXene (0.1 wt%, 0.5 wt% and 1 wt%) composite electrospun fibers. (*P < 0.05).

Bovine serum albumin (BSA) was used as a model protein to test the protein adsorption capacity of different materials. Figure 8 shows the amount of protein adsorbed by each group of materials over 24 h. The results showed that the protein adsorption rate of each nanofiber membrane group was significantly higher than that of the control group, indicating that both SF and MXene promoted the adsorption of proteins. With the addition of mxene nanoparticles, the protein adsorption rate of SF-MXene composite fiber materials also increased, and the protein adsorption rate of SFM1 group was significantly higher than that of SF and SFM0.1 groups. This outcome is likely due to the addition of Ti_3_C_2_Tx MXene, which provides various functional groups that offer more active binding sites for protein adhesion. The increased binding capacity of the fiber membranes for proteins is enhanced through electrostatic interactions, hydrogen bonding, and π-π interactions. Studies have shown that cell surfaces have numerous receptors that interact with proteins, which play a crucial role in the cellular response process (Regis et al., 2014). The excellent protein adsorption capability of the material allows for the rapid formation of a protein layer on its surface, providing more binding sites and thus enhancing cell adhesion and migration. Therefore, the increased protein adsorption capacity of the material significantly enhances its bioactivity. Our study results demonstrate that incorporating Ti_3_C_2_Tx MXene can significantly improve the protein binding ability of SF. This characteristic can also effectively facilitate the binding of scaffold materials with osteoinductive factors such as growth factors and drugs, thereby increasing the material’s application value.

### 3.4 Biomineralization

**FIGURE 9:**
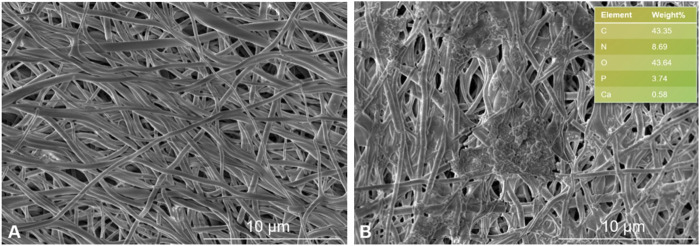
FESEM image of electrospun fibers incubated in SBF solution for 2 weeks: SF **(A)** and SF-Mxene (1 wt%) composite electrospun fibers and EDS (top right) **(B)**.

The mineralization capability of SF-MXene composite fibers was evaluated using an *in vitro* biomineralization assay. SF fibers are well-known matrices for tissue engineering due to their good biocompatibility; it has not yet been evaluated whether composites containing Ti_3_C_2_Tx MXene mineralize. Therefore, the study investigated SF-MXene composites to evaluate their ability to adsorb calcium phosphate minerals in SBF solution. After 14 days of soaking in SBF solution, FESEM images of electrospun SF and SF-MXene composite electrospun fibers are shown in Figure 9. As expected, the SF-MXene composite fibers successfully deposited calcium phosphate minerals (Figure 9B). The deposition of calcium and phosphate ions on the electrospun fiber membrane may be due to the high hydrophilicity of MXene (Joshi et al., 2015). In contrast, no significant calcium phosphate mineral deposition was observed on the pure SF electrospun fibers after 14 days of soaking in SBF solution (Figure 9A). Further confirmation of the presence of calcium and phosphorus in the SBF-treated SF-MXene fiber scaffolds was obtained through EDS analysis. The results demonstrated that the substantial *in vitro* biomineralization and calcium/phosphorus deposition on the SF-MXene composite scaffolds endowed them with sufficient bioactivity, indicating their potential for further application in bone tissue engineering.

### 3.5 Biocompatibility

**FIGURE 10:**
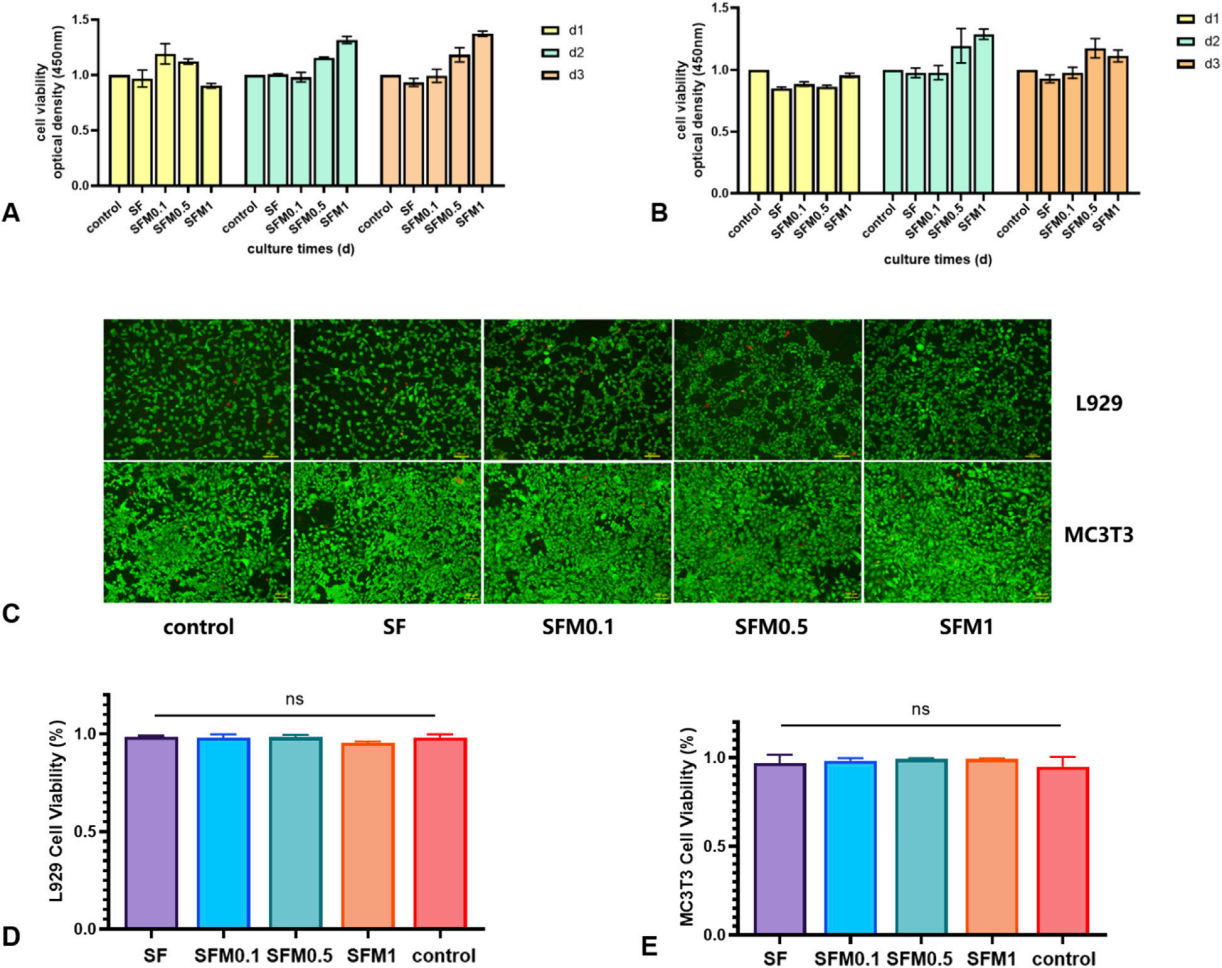
Biocompatibility test. Cell viability test CCK assay **(A, B)**, SF fiber and SF-MXene composite fiber after 3 days of cell culture respectively **(C)**, and cell viability test live and die staining fluorescence ratio determination **(D, E)**.

To determine how SF-MXene composite electrostatic spinning fibers respond to cells, fibroblasts (L929) and pre-osteoblasts (MC3T3-E1) were co-cultured with different electrospun fibers for 1, 2, and 3 days. The biocompatibility of the fiber scaffolds was assessed using the CCK-8 assay and live/dead cell staining. Cells seeded without fiber membranes served as the control group. Figure 10A shows the viability of L929 cells cultured on the electrospun fiber scaffolds for 1, 2, and 3 days. The viability of L929 cells on both SF and SF-MXene composite fibers remained at a high level. Figure 10B shows the cell viability of pre-osteoblasts (MC3T3-E1) cultured on electrospun fibers on the same days. For MC3T3-E1 cells, the viability on both SF and SF-MXene composite fibers with different MXene contents also remained at a high level. Figure 10C shows live/dead cell staining on the third day to determine the viability of L929 and MC3T3-E1 cells on different electrospun fiber membranes. The images from the live/dead staining were analyzed with ImageJ software to determine the ratio of red to green fluorescence, which was used to assess the cell viability on each group of fiber membranes. Figures 10D, E further indicate that the nanofiber electrospun membranes did not exhibit cytotoxicity to L929 cells, as all groups maintained high cell viability. This finding is consistent with the previous CCK-8 assay results.

### 3.6 Alkaline phosphatase (ALP) activity

**FIGURE 11:**
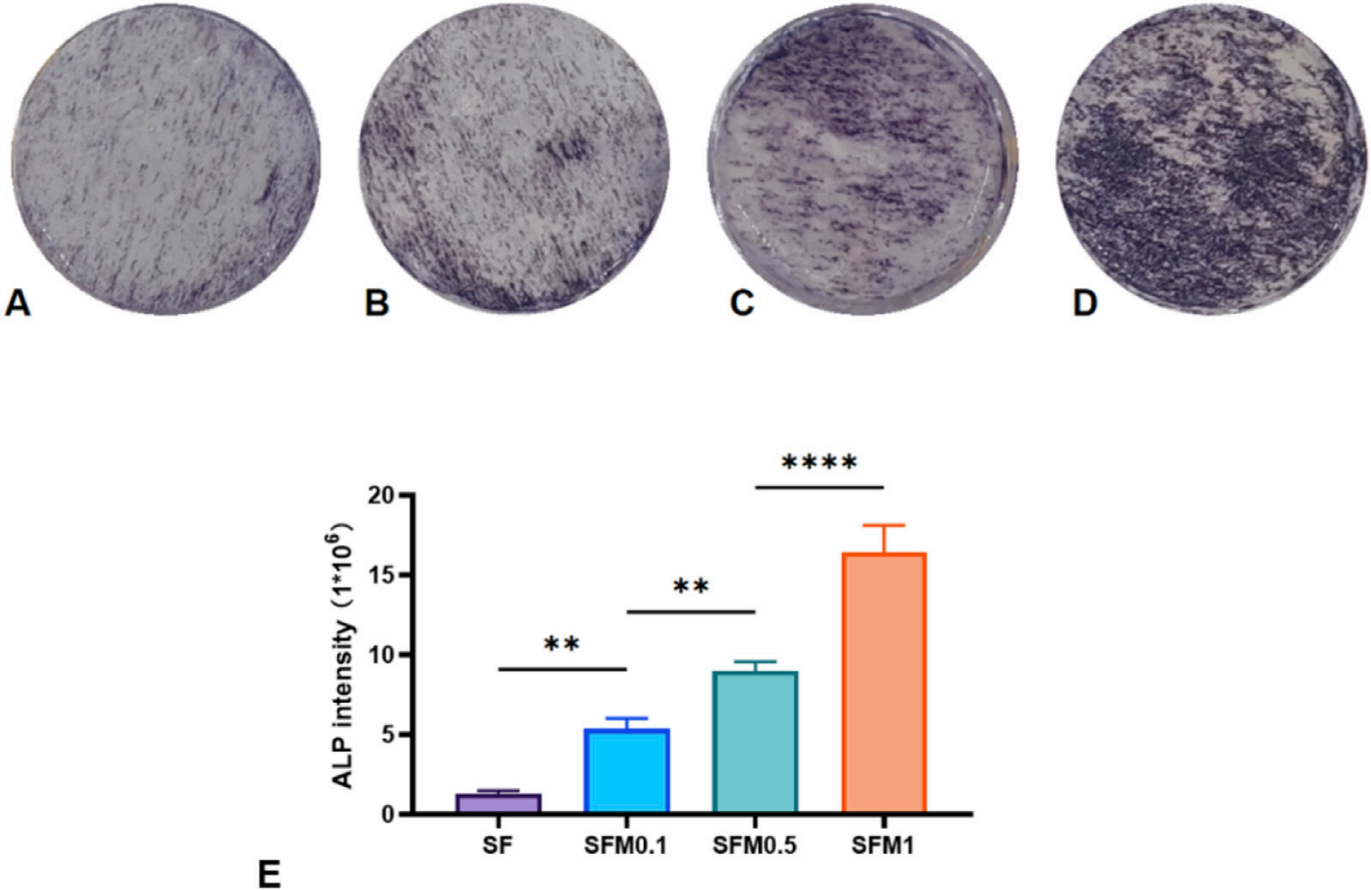
ALP staining was performed after 7 days of co-culture with SF fiber and SF-MXene composite fiber **(A–D)**. **(E)** Quantitative analysis of ALP staining. (**P < 0.01, ***P < 0.001, ****P < 0.0001).

Alkaline phosphatase (ALP) is crucial in bone formation and mineral deposition; thus, assessing ALP staining intensity can provide an indirect measure of cellular mineralization capability and mineral deposition. In this study, the ALP activity was examined by culturing MC3T3-E1 cells on nanofiber membranes with varying amounts of MXene, followed by ALP staining after 7 days of culture. As illustrated in Figure 11A, cells cultured on pure SF exhibited lower ALP intensity, while increasing MXene content led to a marked enhancement in ALP staining intensity (Figures 11B–D), with the highest intensity observed at 1 wt% MXene content. Semi-quantitative analysis of the stained images confirmed that higher MXene content significantly elevated ALP activity, suggesting that MXene promotes early osteoblast differentiation and enhances cellular mineralization and mineral deposition (Figure 11E).

## 4 Conclusion

In this study, we successfully demonstrated the electrospinning of silk fibroin (SF)-MXene composite fibers. The obtained electrospun fibers were characterized for their physicochemical properties and *in vitro* biocompatibility. Our results indicate that both SF fibers and SF-MXene composite fibers exhibit excellent biocompatibility with L929 and MC3T3 cells, underscoring their potential for biomedical applications. Compared to pure SF fiber membranes, the SF-MXene composite fibers displayed notable improvements in wettability, mechanical properties, biomineralization, and protein adsorption. These enhancements not only improve the fibers’ biocompatibility but may also broaden their applicability in tissue engineering and regenerative medicine. We attribute the unique properties of SF-MXene composite fibers to the abundance of active hydrophilic groups on the MXene surface, which bind effectively to the amino acid residues on the SF surface. Furthermore, MXene itself possesses valuable properties, including hydrophilicity, non-toxicity, and the ability to promote cell proliferation and biomineralization. These composite electrospun fibers show promise for biomedical applications such as wound dressings and bone tissue engineering. Additionally, the approach presented here provides a theoretical basis for the development of MXene-based polymer fiber composites, supporting future applications in tissue regeneration engineering.

## Data Availability

The raw data supporting the conclusions of this article will be made available by the authors, without undue reservation.
